# Effect of total sleep deprivation on effective EEG connectivity for young male in resting-state networks in different eye states

**DOI:** 10.3389/fnins.2023.1204457

**Published:** 2023-10-19

**Authors:** Mengke Ma, Yutong Li, Yongcong Shao, Xiechuan Weng

**Affiliations:** ^1^School of Psychology, Beijing Sport University, Beijing, China; ^2^Key Laboratory for Biomechanics and Mechanobiology of the Ministry of Education, School of Biological Science and Medical Engineering, Beihang University, Beijing, China; ^3^Department of Neuroscience, Beijing Institute of Basic Medical Sciences, Beijing, China

**Keywords:** resting-state, electroencephalogram, total sleep deprivation, neural oscillation, effective connectivity

## Abstract

**Background:**

Many studies have investigated the effect of total sleep deprivation (TSD) on resting-state functional networks, especially the default mode network (DMN) and sensorimotor network (SMN), using functional connectivity. While it is known that the activities of these networks differ based on eye state, it remains unclear how TSD affects them in different eye states. Therefore, we aimed to examine the effect of TSD on DMN and SMN in different eye states using effective functional connectivity via isolated effective coherence (iCoh) in exact low-resolution brain electromagnetic tomography (eLORETA).

**Methods:**

Resting-state electroencephalogram (EEG) signals were collected from 24 male college students, and each participant completed a psychomotor vigilance task (PVT) while behavioral data were acquired. Each participant underwent 36-h TSD, and the data were acquired in two sleep-deprivation times (rested wakefulness, RW: 0 h; and TSD: 36 h) and two eye states (eyes closed, EC; and eyes open, EO). Changes in neural oscillations and effective connectivity were compared based on paired *t*-test.

**Results:**

The behavioral results showed that PVT reaction time was significantly longer in TSD compared with that of RW. The EEG results showed that in the EO state, the activity of high-frequency bands in the DMN and SMN were enhanced compared to those of the EC state. Furthermore, when compared with the DMN and SMN of RW, in TSD, the activity of DMN was decreased, and SMN was increased. Moreover, the changed effective connectivity in the DMN and SMN after TSD was positively correlated with an increased PVT reaction time. In addition, the effective connectivity in the different network (EO-EC) of the SMN was reduced in the β band after TSD compared with that of RW.

**Conclusion:**

These findings indicate that TSD impairs alertness and sensory information input in the SMN to a greater extent in an EO than in an EC state.

## Introduction

Exploring resting-state brain activity has attracted increased attention since Hans Berger first used an electroencephalogram (EEG) to observe the human brain’s activity during rested wakefulness (RW) (Berger, 1930/1969; Berger, 1931/1969). Resting-state brain activity reflects spontaneous cognition associated with self-referential meditation (Kozasa et al., 2017), mind wandering (Zhou and Lei, 2018), and context setting for future information processing (Raichle, 2006). Furthermore, an increasing amount of literature suggests that resting-state brain activity is linked to many disorders, including Alzheimer’s disease (Snyder et al., 2011; Chiang and Pao, 2016; Babiloni et al., 2020), Parkinson’s disease (Wang et al., 2018), and epilepsy (Park et al., 2018).

Generally, resting-state data are acquired in different eye states, including eyes-closed (EC), eyes-open (EO), and EO with visual fixation states. Several studies have demonstrated that resting-state activity patterns are different in different eye states. For example, Chen et al. (2008) observed significant differences between EC and EO states in almost all EEG power spectrum frequency bands. Furthermore, Liu et al. (2013) found that the differences between EO and EC states were repeatable across three different calculating methods, namely amplitude of low-frequency fluctuation (ALFF), regional homogeneity, and seed-based correlation analysis. This implies that there are robust differences in the activity between EC and EO states.

In the resting state, the default mode network is the most active network; it is also the most widely studied network for differences between EC and EO states. It is largely activated during stimulus-free states and deactivated during externally oriented tasks (Raichle and Snyder, 2007; Satpute and Lindquist, 2019). It includes several functional core regions, including the precuneus (PCu), medial prefrontal cortex, posterior cingulate cortex (PCC), and inferior parietal lobule (IPL) (Shirer et al., 2012). The DMN is also involved in collecting and evaluating information (Gusnard and Raichle, 2001), daydreaming (Christoff et al., 2009; Kucyi et al., 2013), emotion (Simpson et al., 2001), and extracting episodic memory (Cabeza et al., 2002). Some investigations have indicated that the functional connectivity (FC) of the DMN was similar across different eye states (Fox et al., 2005; Fransson, 2005; Patriat et al., 2013). However, many studies had opposite conclusions. For example, Yan et al. (2009) observed that the EO state induced significantly higher FC and ALFF of the DMN than those induced by the EC state. Furthermore, Van Dijk et al. (2010) reported that FC in the DMN was higher during visual fixation than during the EC state. In addition, Liu et al. (2020) observed that the static FC of the DMN in the EC state was similar to that in the EO state; However, they identified three group-level dynamic FC states (States 1, 2, and 3), and the strength of dynamic FC differed among EC and EO in States 2 and 3. Specifically, the dynamic FC was lower in State 2 of the EC than it was in the EO. Conversely, the dynamic FC was higher in State 3 of the EC than in that of the EO. This reminds us that the internal mental activity of participants is constantly changing with time, which may cause the FC of the DMN to change with time. In conclusion, the conflicting results of the above studies on the DMN may be due to uncontrollable internal mental activity of participants as well as the different research methods used.

In addition to states in the DMN, differences between EO and EC states in other brain regions have also been extensively studied. For example, Marx et al. (2004) observed that blood oxygenation level-dependent signals were lower in cortical areas related to somatosensation in the EO state than in the EC state, which was considered an interoceptive state (Marx et al., 2003). Furthermore, Yang et al. (2007) reported that the EO state had higher ALFF values in the bilateral visual cortices and lower ALFF values in the right paracentral lobule than the EC state. After that, Yuan et al. (2014) observed decreased amplitude of fluctuation (AF) of both the high- and low-frequency bands in the primary auditory and primary sensorimotor cortices and increased AF in the visual cortex in the EO state than in the EC state. Additionally, Xu et al. (2014) observed that the EC state had higher regional nodal properties in multiple sensory systems than the EO state. The research results above show that the activity in the sensorimotor cortex is increased in the EC state than the EO state. Furthermore, these similar results indicate a more robust different pattern between the EC and EO states in the SMN than in the DMN.

Sleep deprivation is a widely used paradigm for studying the effects of sleep on vigilance, cognitive functions, memory consolidation, etc. Several studies have indicated that SD impairs multiple cognitive functions (Chee and Chuah, 2007; Simon et al., 2015; Lei et al., 2016), including causing declines in FC within the DMN (De Havas et al., 2012; Lei et al., 2015; Yang et al., 2018), which has been well demonstrated to be associated with many higher cognitive functions (Bembich et al., 2014; Charroud et al., 2015; Japee et al., 2015). However, previous studies collected data only in the EO or EC states separately, and the effect of SD on FC in the DMN under different eye conditions has rarely been investigated. Some special populations, such as drivers, doctors, pilots, and soldiers, often have to carry out intensive work under SD, during which their eyes need to be open. Therefore, studies on the influence of total SD (TSD) on resting with EO have wider practical significance than those on the EC state. Although persons under SD are extremely sleepy, they still try to stay awake. In this situation, the EC state is more like a state of microsleep. Microsleep is a state in the wake–sleep transition zone that is characterized by rapid fluctuations between wakefulness and sleep (Hertig-Godeschalk et al., 2020). Microsleep is defined as an intrusion of theta rhythms in EEG, lasting between 3 and 14 s (Morrone et al., 2019). Microsleep is more likely to occur when eyes are closed than when eyes are open. So, the EO state may better reflect an antagonistic process of individuals to sleepiness. Therefore, the EO state can better reflect the effect of TSD on cognitive functions than the EC state. Nevertheless, in prior studies on the influence of TSD on cognitive function, resting-state data were acquired in the EO or EC state, and there was no uniform standard regarding the state of the eyes. Hence, it is valuable to investigate the different effects of TSD on resting brain activity in different eye states.

Prior studies detecting the EO-EC difference mostly used the static or dynamic FC based on functional magnetic resonance imaging (fMRI) with a lower temporal resolution, which might have caused one-sided results. In comparison, EEG has a much higher temporal resolution and can directly estimate cortical activity (Aoki et al., 2015). Furthermore, exact low-resolution brain electromagnetic tomography (eLORETA), a high-precision brain imaging method, can identify networks across the dimensions of space and frequency (Aoki et al., 2015) as well as directed effective connectivity within networks. Contrastingly, the fMRI networks are detected according to time and space dimensions, whereas FC based on fMRI was undirected. Given these advantages of EEG analysis, we employed eLORETA to calculate the differences in neural oscillation and effective connectivity between the EC and EO states. In addition, we examined how TSD affects the resting-state networks during different eye states.

The study’s hypotheses were as follows: (1) the spontaneous and synchronous activity in the DMN and SMN was different between EC and EO states; (2) the spontaneous and synchronous activity of DMN and SMN was different after TSD compared with those after RW; and (3) the different network (EO-EC) of DMN and SMN was different between TSD and RW. Because of the difference in brain activity between open and closed eyes states, different eye states may be an additional variable in many resting state studies. The goal of the study was to explore the effects of different eye states on brain activity. In addition, we also introduced the variable TSD to provide guidance for the selection of eye states during the collection of resting-state data in sleep deprivation.

## Materials and methods

### Participants

We recruited 24 male students (mean age, 22 years; range, 21–25 years; standard deviation, 1.39), and five were excluded from the statistical analysis because of technical problems and data loss. Therefore, 19 participants were included in the data analysis. The inclusion criteria were as follows: (1) healthy young male with normal uncorrected or corrected vision; (2) right-handed; (3) no use of medications or drugs, including psychotropic drugs, sleeping pills, cold medicines, etc., in the 2 weeks before the experiment; (4) no serious medical history such as hepatitis, cancer, nephritis, diabetes, or endocrine disorders; (5) no history of psychiatric diseases; (6) no habit of drinking coffee, tea, or smoking; (7) had not taken caffeinated or alcoholic beverages and had not exercised vigorously for 2 weeks prior to the experiment; (8) Pittsburgh Sleep Quality Index Questionnaire (Buysse et al., 1989) scores before the experiment <5; and (9) did not participate in psychological and physiological tests before. All participants signed written informed consent prior to the experiment, and our study was approved by the Biological and Medical Ethics Committee of Beihang University (ethics code: BM20180040).

### Experimental procedures

**Figure 1:**
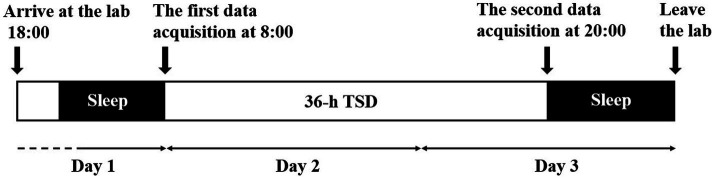
Flowchart of the sleep deprivation experiment. Participants arrived at 18:00 on the first day and slept in the laboratory for the first night of the experiment. The first data acquisition was performed at 8:00 on the second day, including the resting-state EEG task and PVT. The second data acquisition was performed at 18:00 on the third day. Participants underwent a recovery sleep and left the lab subsequently.

Participants were asked to report their bedtime and waking times each day for 2 weeks prior to the experiment to ensure a regular sleep duration of 7–9 h. They arrived at the laboratory at 18:00 on the first day of the study period (Figure 1). Then, they were informed of the procedure and risks of the experiment and then signed a written informed consent form. The participants were asked to sleep in our laboratory for the night prior to the formal experiment. The formal experiment began at 8:00 the next day. Each participant underwent 36 h of TSD, and all the data were acquired in four experimental conditions [two eye states (EC; EO) and two sleep deprivation times (RW: 0 h; TSD: 36 h)]. Participants were asked to sit quietly and relax for 2 min upon wearing the EEG equipment. All the participants underwent the resting-state EEG tasks both in EC and EO states, and the different eye states were counterbalanced. Twelve participants were asked to keep their eyes open for the first 5 min, while the other twelve kept their eyes closed for the first 5 min, with a 2-min break in between.

They completed the first resting-state EEG task and PVT successively, meanwhile, behavioral and EEG data were recorded. After TSD (at 20:00 on the third day), the second resting-state EEG task and PVT were completed. During the whole experiment time, participants were allowed to have meals only at 7:30, 12:00, and 17:30. In the rest of the experimental period, participants could conduct their personal affairs. Simultaneously, they were supervised by our main staff to prevent them from napping. Meanwhile, vigorous exercise and high-arousal activities were forbidden, and they could not leave the laboratory. When completing the experiment, participants had recovery sleep in our laboratory and left the laboratory subsequently.

### PVT

The PVT is highly sensitive to SD and has become the most widely used measurement approach for behavioral vigilance (Dorrian et al., 2005; Lim and Dinges, 2008). Thus, it was selected to indicate the participants’ psychomotor vigilance levels. At the beginning of each trial, the symbol “+” appeared for 400 ms at the center of a white background on the display screen (1,024 × 768-pixel resolution; refresh rate, 60 Hz). Then, red dots (diameter: 3 cm; viewing angle: 1.5 × 1.5°) appeared at the center of the screen for 1,000 ms and immediately disappeared when the participants pressed the button. The intervals between the trials were 8–12 s (mean: 10 s) and were pseudorandomized. Participants were asked to react as soon as a red dot appeared; however, early responses were not allowed. There were 30 trials in total; reaction times (RTs) to trials ≤150 ms were defined as an early response, and RTs ≥550 ms were defined as a lapse.

### EEG recording

The EEG data were recorded based on the international standard 10–20-electrode-placement system using Neuroscan 32-channel electrode caps combined with a Syn-Amps2 amplifier (Compumedics Neuroscan, Charlotte, NC, USA) at a 1000-Hz sampling rate. All electrode impedances were < 5 kΩ prior to recording. Two electrodes were placed outside both eyes to record the horizontal electrooculogram, and another two electrodes were placed on the upper and lower sides of the left eye to record the vertical electrooculogram. The data of five participants were excluded from the statistical analysis due to data loss. Hence, the final sample comprised 19 participants.

### EEG data preprocessing

The EEGLAB toolbox in MATLAB R2020B software (Mathworks, Natick, MA, USA) was used to preprocess the raw EEG signals offline.

1. We reduced the sampling rate to 250 Hz.
2. A band-pass filter (0.1–40 Hz) was used via the Hamming windowed sinc FIR filter (transition bandwidth: 2 Hz, order: 414).
3. We manually removed the data segments with obvious artifacts. Bad channels were replaced with interpolation methods, and 0.63 bad channels per participant were replaced, on average.
4. Independent component analysis (ICA) was applied to identify independent components with stereotypical artifacts. Subsequently, an average of 6.1 components were manually removed per participant.
5. Artifact detection was performed with voltage limits of −100 ~ 100 μV.
6. The whole-brain average reference was set.
7. After preprocessing, EEG data without artifacts were acquired. Subsequently, the functional independent component and effective connectivity analysis were performed.

### EEG source reconstruction method

In this study, we employed eLORETA to generate the estimated cortical electrical distribution from scalp signals because scalp signals cannot be directly used to compute cortical connections (Pascual-Marqui and Biscay-Lirio, 2011). The EEG signal is formed by the firing of neurons, and it reaches the scalp electrode through the tissue media such as cerebrospinal fluid and skull. Scalp potentials are affected by volume conduction effects so that electrodes at a given scalp location can detect the activity of neurons in the area directly below them as well as the activity of a remote source. The activation of one source simultaneously affects all scalp electrodes, resulting in an intrinsic correlation between the signals recorded on these electrodes. Therefore, signals acquired on scalps cannot be directly used to compute cortical connections, and the analysis of recorded EEG signals at different locations to determine the source of their generation is referred to as source localization analysis.

We opted to use eLORETA because it is an improved version of both LORETA and standardized LORETA (Pascual-Marqui, 2002). According to Pascual-Marqui, eLORETA is a weighted minimum norm inverse solution that provides exact localization with zero error, even in the presence of measurement and structured biological noise (Pascual-Marqui, 2007). The solution space comprised 6239 cortical gray matter voxels at a spatial resolution of 5 mm in a realistic head model (Fuchs et al., 2002). The Montreal Neurologic Institute (MNI) 152 template was used with Brodmann area (BAs) anatomic labels (Mazziotta et al., 2001).

### Functional independent component analysis

Through functional independent component analysis, we aimed to examine the changes of spontaneous brain activity. Resting-state EEG data were analyzed by functional independent component analysis (fICA) using eLORETA to compute changes in neural oscillations (Pascual-Marqui and Biscay-Lirio, 2011). Cortical electrical activity was calculated for the following frequency bands: 1 Hz < δ ≤ 4 Hz; 4 Hz < θ ≤ 8 Hz; 8 Hz < α ≤ 12 Hz; 12 Hz < β ≤ 30 Hz; 30 Hz < γ ≤ 40 Hz. EEG data were converted into frequency domain to generate a set of cross spectral indicators using discrete Fourier transform. Subsequently, the spectral density of each frequency band and cortical voxel was calculated. Networks were divided according to the similarity of signals, in which similar signals were assigned to the same network, and the dissimilar signals were divided into different networks. Therefore, we obtained five networks. There were five coefficients corresponding to the five networks, with each representing a different frequency band (δ, θ, α, β, and γ). It is important to note that the cortical electrical distribution of this network was quite different from that of known functional networks (Chen et al., 2013). Functional networks deriving from fICA were data-driven, and the figures exported by eLORETA were not presented as a known whole functional network, and the brain regions of DMN and SMN might be mixed. Next, each participant’s data were linked to produce a matrix with dimensions of spatial frequency and different participants (Aoki et al., 2015).

Subsequently, the five coefficients were statistically analyzed to compare the differences in the functional networks of the resting states between different eye states or different SD times. Paired *t*-tests w ere applied to test RW (EO) = RW (EC), TSD (EO) = TSD (EC), TSD (EO) = RW (EO), TSD (EC) = RW (EC), and TSD (EO-EC) = RW (EO-EC). Simultaneously, randomization statistical nonparametric mapping (SnPM) was used to correct multiple comparisons (Thatcher et al., 2005; Painold et al., 2020). Finally, the functional networks with significant differences were identified, which were represented by independent components (ICs). They were permitted to have minor differences and overlap with traditional functional networks. They were not presented as a typical DMN or SMN, and the changed activity in each frequency band was presented as a network.

### Effective connectivity analysis

Functional connectivity has been extensively used to analyze functional networks. As a special functional connectivity, the effective connectivity provides direction information regarding the connections between different brain regions. We employed the effective connectivity analysis to assess the changes in synchronous activity in different conditions. Effective connectivity analysis was performed using eLORETA software with the isolated effective coherence (iCoh) algorithm. The definition of iCoh is based on formulating a multivariate autoregressive model and calculating the corresponding partial coherences after setting all irrelevant connections to zero other than the particular directional association of interest (Pascual-Marqui et al., 2014). We aimed to assess the direct paths of intracortical causal information flow of oscillatory activity within the SMN and DMN and the related changes between different eye states or different SD times. First, we selected brain regions in the SMN and DMN as the regions of interest (ROIs) by referring to previous studies and the results of fICA (Table 1). Then, iCoh values were calculated for 1–40 Hz, corresponding to five frequency bands: 1 Hz < δ ≤ 4 Hz; 4 Hz < θ ≤ 8 Hz; 8 Hz < α ≤ 12 Hz; 12 Hz < β ≤ 30 Hz; 30 Hz < γ ≤ 40 Hz. Paired *t*-tests were used to test RW (EO) = RW (EC), TSD (EO) = TSD (EC), TSD (EO) = RW (EO), TSD (EC) = RW (EC), and TSD (EO-EC) = RW (EO-EC). Randomization SnPM was used (number of randomizations: 5000) for the multiple comparison correction (Painold et al., 2020), then we acquired the corrected critical thresholds and *p*-values.

**Table 1:** Montreal Neurological Institute (MNI) region of interest coordinates in the sensorimotor and default mode networks.

Structure	Regions of Interest (ROI)	Brodmann area	Side	*X*	*Y*	*Z*
Frontal Lobe	L. Precentral Gyrus	4	L	−35	−30	70
Frontal Lobe	R. Precentral Gyrus	4	R	35	−30	70
Frontal Lobe	L. Superior Frontal Gyrus	6	L	−10	−20	70
Frontal Lobe	R. Superior Frontal Gyrus	6	R	10	−20	70
Parietal Lobe	L. Superior Parietal Lobule	7	L	−30	−60	65
Parietal Lobe	R. Superior Parietal Lobule	7	R	30	−60	65
Parietal Lobe	L. Postcentral Gyrus	7	L	−25	−55	70
Parietal Lobe	R. Postcentral Gyrus	7	R	25	−55	70
Parietal Lobe	L. Precuneus	7	L	−10	−65	65
Parietal Lobe	R. Precuneus	7	R	10	−65	65
Parietal Lobe	L. Inferior Parietal Lobule	40	L	−40	−55	60
Parietal Lobe	R. Inferior Parietal Lobule	40	R	40	−55	60
Frontal Lobe	L. Medial Frontal Gyrus	6	L	−10	−30	70
Frontal Lobe	R. Medial Frontal Gyrus	6	R	10	−30	70
Limbic Lobe	L. Posterior Cingulate Cortex	31	L	−3	−57	21
Limbic Lobe	R. Posterior Cingulate Cortex	31	R	3	−57	21

### Statistical analysis

We used SPSS software (version 26.0) to perform paired *t*-tests on the PVT parameters between RW and TSD. Pearson’s correlation analysis was used on the changes in significant effective connectivity and PVT parameters before and after TSD. The level of statistical significance was set at *p* < 0.05. We corrected multiple comparisons using the False Discovery Rate (FDR). We chose Pearson’s correlation because it was the most common and effective way to measure a linear relationship between two continuous variables. Examining the correlations between effective connectivity and PVT response time could better illustrate the physiological significance of effective connectivity.

We chose paired *t*-tests as our statistical method for the fICA and iCoh analyses because we were not concerned with the interaction effect between our two independent variables; we were only concerned with the comparison between two states (EC and EO; RW and TSD). Moreover, after the *t*-test, we had strictly corrected the value of *p* to rule out false positive results by using SnPM. As our statistical method was able to verify the research hypotheses, we did not use analysis of variance.

## Results

### PVT results

The PVT parameters of the 19 participants differed significantly between the RW and TSD groups (Table 2). The paired *t*-test results suggested that compared with RW, the mean RT was significantly longer after TSD (*t_18_* = 2.31, *P_FDR-corrected_* = 0.041). Furthermore, the median RT was significantly longer (*t_18_* = 3.07, *P_FDR-corrected_* = 0.023), the fastest 10% RT was significantly slower (*t_18_* = 2.15, *P_FDR-corrected_* = 0.045), the lowest 10% RT was significantly prolonged (*t_18_* = 2.37, *P_FDR-corrected_* = 0.041), and the number of lapses was significantly higher (*t_18_* = 2.91, *P_FDR-corrected_* = 0.023) after TSD. We corrected multiple comparisons using False Discovery Rate (FDR).

**Table 2:** Changes of PVT indicators.

Behavioral metrics	RW	TSD	*t*	*P_FDR-corrected_*	Cohen’s d
Mean RT (ms)	294.33 ± 27.95	309.63 ± 44.53	2.31	0.041	0.529
Median RT (ms)	277.55 ± 27.96	296.13 ± 41.51	3.07	0.023	0.705
Fastest 10% RT (ms)	228.26 ± 22.82	236.56 ± 27.16	2.15	0.045	0.494
Lowest 10% RT (ms)	464.46 ± 90.34	666.74 ± 352.74	2.37	0.041	0.545
Number of Lapses	0.32 ± 0.478	1.63 ± 1.98	2.91	0.023	0.667

Data are presented as the mean ± standard deviation. RW, rested wakefulness; TSD, total sleep deprivation; RT, reaction time.

### Neural oscillation changes between EC and EO states in RW

**Figure 2:**
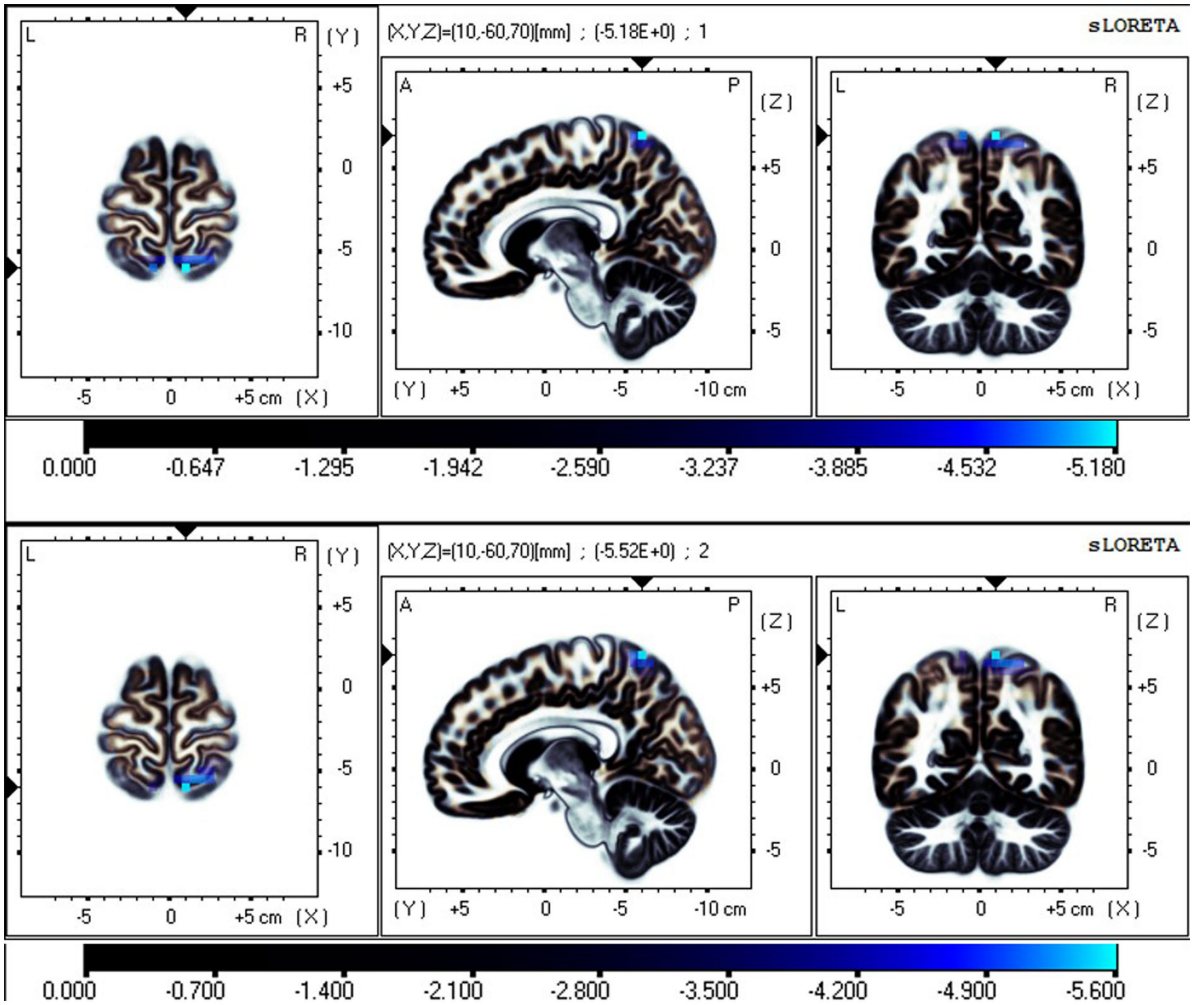
Neural oscillation changes in RW between EC and EO states, IC2 (*t-corrected* = 3.602, *p-corrected* < 0.001). From top to bottom: δ and θ bands. Significant areas: the postcentral gyrus (BA7 and 5), superior parietal lobule (BA7), and precuneus (BA7). The color bar represents the *t* values, and bright blue represents oscillation power decrease with increasing *t* values (EO-EC). BA, Brodmann area; L, left; R, right; A, anterior; P, posterior.

**Figure 3:**
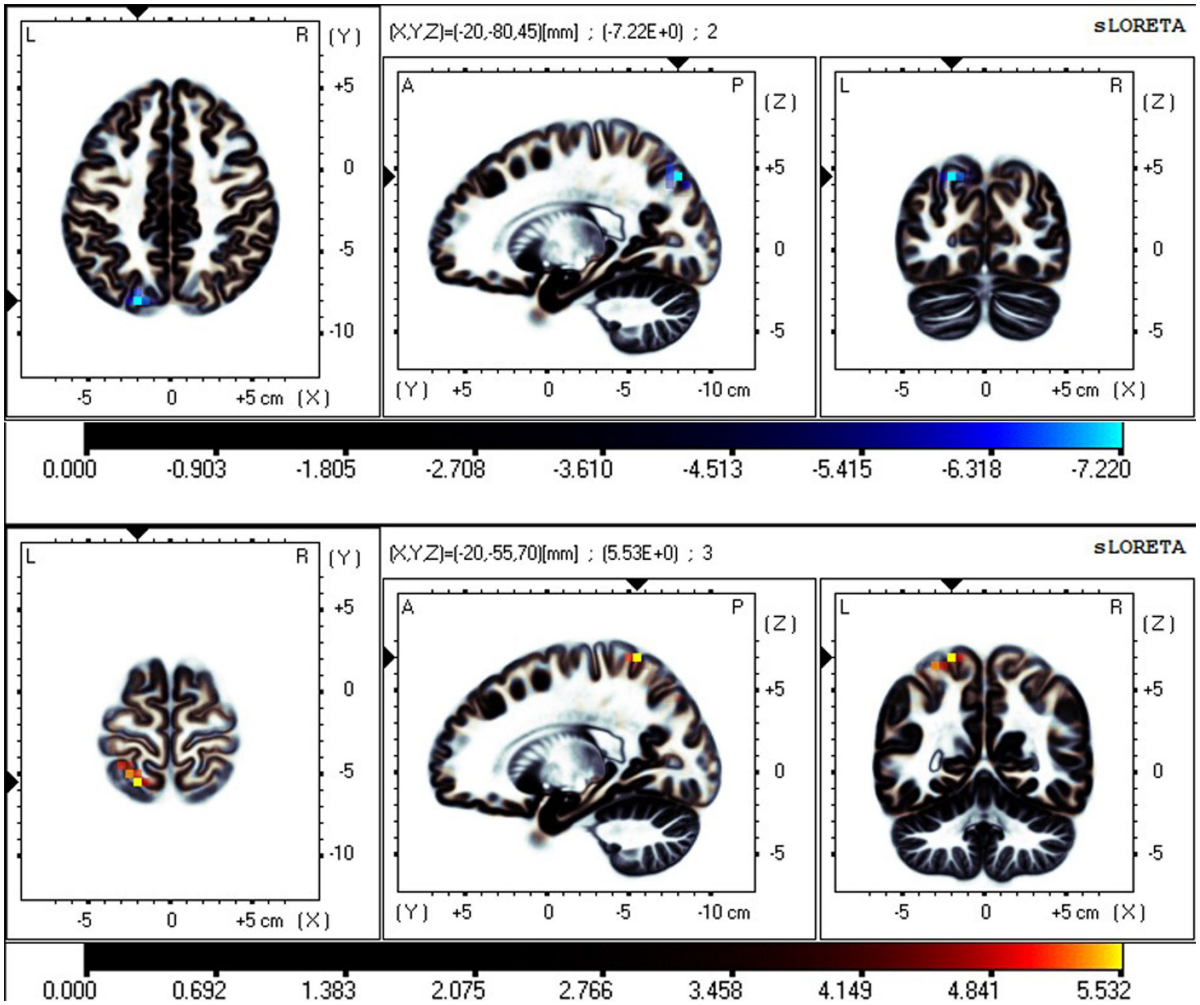
Neural oscillation changes in RW between EC and EO states, IC5 (*t-corrected* = 4.485, *p-corrected* < 0.001). From top to bottom: θ and α bands. IC5-θ Significant areas: the precuneus (BA7 and 19) and superior parietal lobule (BA7). IC5-α significant areas: the postcentral gyrus (BA7 and 5) and superior parietal lobule (BA7). Bright blue represents oscillation power decrease with increasing *t* values (EO-EC). Bright yellow represents oscillation power increase with increasing *t* values (EO-EC).

**Figure 4:**
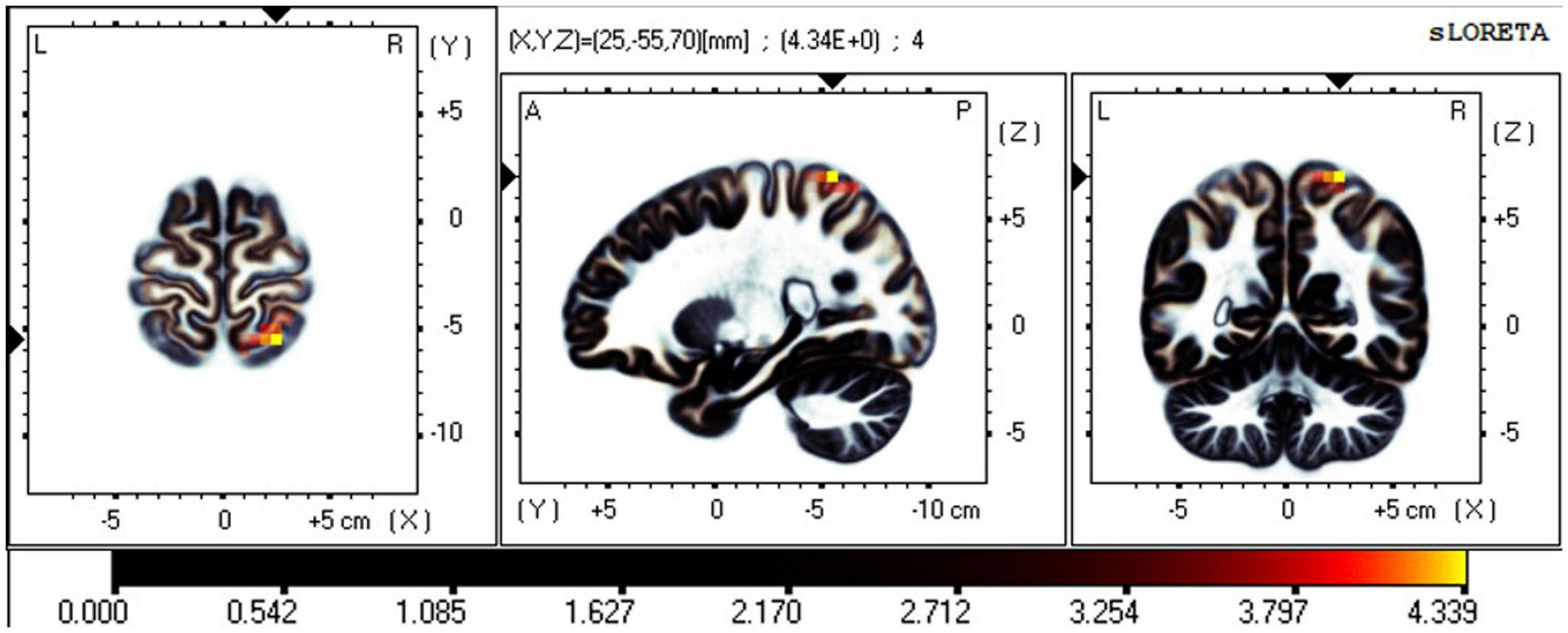
Neural oscillation changes in RW between EC and EO states, IC6 (*t-corrected* = 3.513, *p-corrected* < 0.001). IC6-β significant areas: the postcentral gyrus (BA7, 5) and superior parietal lobule (BA7).

The fICA results showed that the independent components (ICs), representing data-driven functional network, significantly differed in the EC and EO states. The neural oscillations in the SMN were significantly different in the EO state compared to the EC state. This was manifested in the postcentral gyrus and superior parietal lobule, with reduced neural oscillations in the δ and θ bands and enhanced oscillations in the α and β bands in the EO state (Figures 2–4). Additionally, the neural oscillations in the DMN were significantly decreased in the EO state compared to the EC state. This was reflected in the PCu in the δ and θ bands (Figures 2, 3).

### Neural oscillation changes between RW and TSD in the EC state

**Figure 5:**
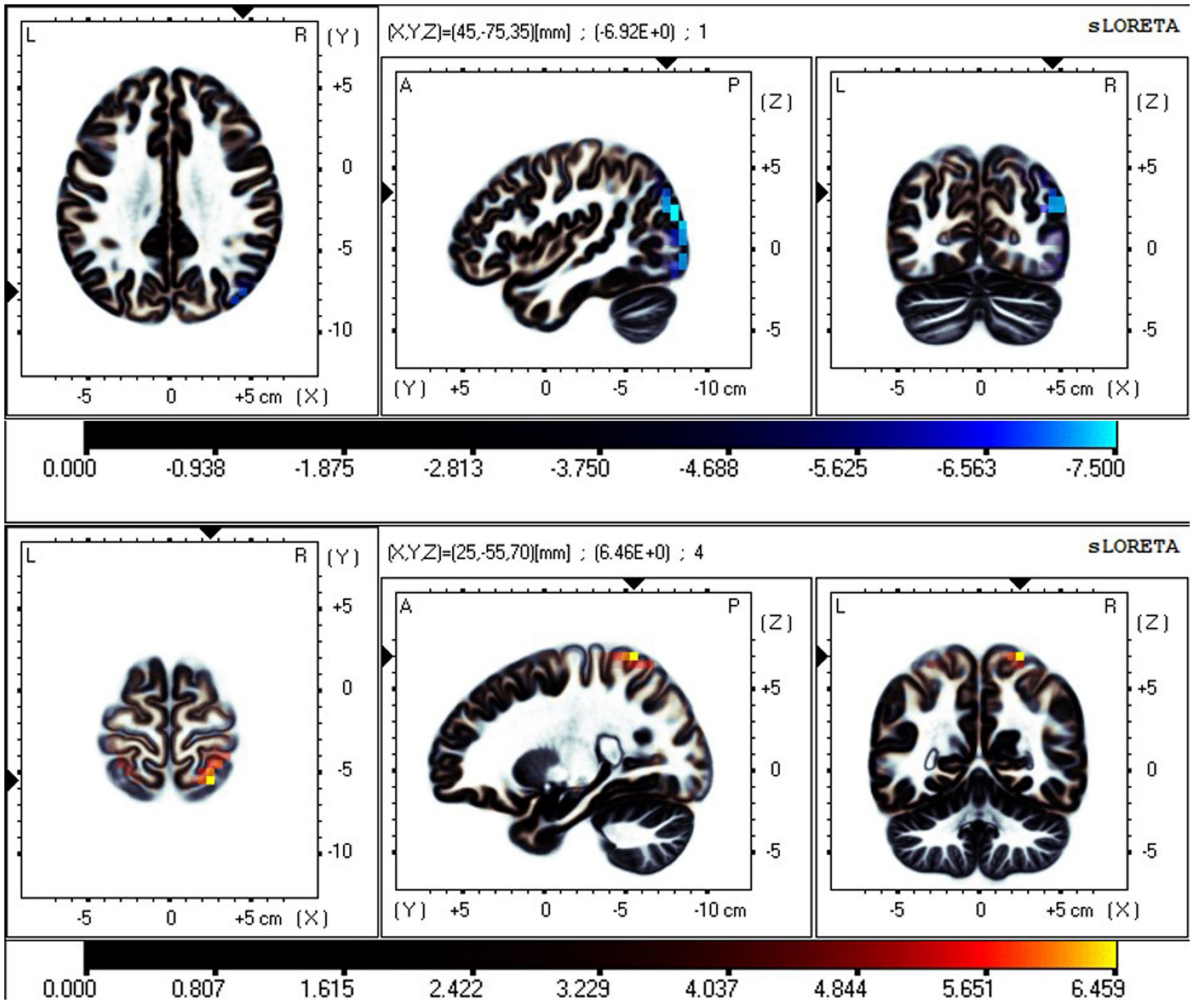
Neural oscillation changes between RW and TSD in the EC state. From top to bottom: IC1-δ (*t-corrected* = 0.594, *p-corrected* < 0.001) and IC7-β (*t-corrected* = 1.429, *p-corrected* = 0.035). IC1-δ significant areas: the precuneus (BA 39, 19, 7). IC7-β significant areas: the postcentral gyrus (BA7, 5, 2, 1, 3, and 40), superior parietal lobule (BA7), and precentral gyrus (BA4 and 6).

The fICA results showed that in the EC state, two ICs significantly differed between RW and TSD. In the EC state, neural oscillations in the PCu significantly decreased in the δ band in TSD compared with RW (Figure 5). Moreover, neural oscillations of the β band (Figure 5) in the SMN significantly increased, including in the postcentral gyrus (BA7, 5, 2, 1, 3, and 40), superior parietal lobule (BA7), and precentral gyrus (BA4 and 6).

### Effective connectivity changes between the EC and EO states in RW

**Figure 6:**
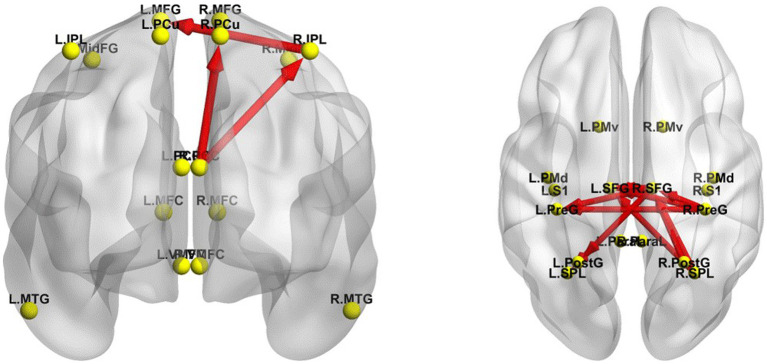
Changes in effective connectivity between EC and EO resting states. Left panel, axial view (dorsal side) of default mode network (DMN). Right panel, coronal view (back side) of sensorimotor network (SMN). Red indicates that the effective connectivity is higher in the EO state than in the EC state. The arrow indicates the direction of the connection. L, left; R, right. PCu, precuneus; IPL, inferior parietal lobule; MFG, medial frontal gyrus; PCC, posterior cingulate cortex; MTG, middle temporal gyrus; MFC, medial frontal cortex; VMFC, ventromedial frontal cortex; MidFG, middle frontal gyrus; PreG, precentral gyrus; SFG, superior frontal gyrus; SPL, superior parietal lobule; PostG, postcentral gyrus; S1, primary sensory cortex; PMd, dorsal premotor cortex; PMv, ventral premotor cortex; ParaL, paracentral lobule. Frequency bands: δ: 1–4 Hz; θ: 4–8 Hz; α: 8–12 Hz; β: 12–30 Hz; γ: 30–40 Hz.

The iCoh results suggested that the effective connectivity in the DMN in the resting-state was significantly higher in the EO state than in the EC state (*t-corrected* = 4.046, *p-corrected* = 0.0016) (Figure 6). The right IPL sent to the left MFG, oscillating at the α and β bands (*t-corrected* = 4.453); the right PCC sent to the right PCu oscillating at the α and β bands (*t-corrected* = 4.926), and sent to the right IPL oscillating at the β and γ bands (*t-corrected* = 5.510).

In addition, the iCoh results indicated that the effective connectivity in the SMN was significantly enhanced in the resting-state with EO compared to that with EC (*t-corrected* = 3.900) (Figure 6). The right SPL sent to the right SFG oscillating at α, β, γ bands (*t-corrected* = 6.097), and to the left SFG at β and γ bands (*t-corrected* = 5.149); the right PreG sent to the right (*t-corrected* = 4.868) and left (*t-corrected* = 4.733) SFG oscillating at α, β and γ bands, and to the left PreG at β and γ bands (*t-corrected* = 4.264); the left SFG sent to the right SFG oscillating at the γ band (*t-corrected* = 4.623); the right SFG sent to the left SPL oscillating at α and β bands (*t-corrected* = 4.449), to the left SFG at β band (*t-corrected* = 4.331), to the left PreG at β and γ bands (*t-corrected* = 4.292), and to the left PostG at α and β bands (*t-corrected* = 4.286); the right PostG sent to the right SFG oscillating at γ band (*t-corrected* = 4.714). Among all the frequency bands, the β band was the most significant for above effective connectivity in DMN and SMN.

### Effective connectivity changes between the EC and EO states in TSD

**Figure 7:**
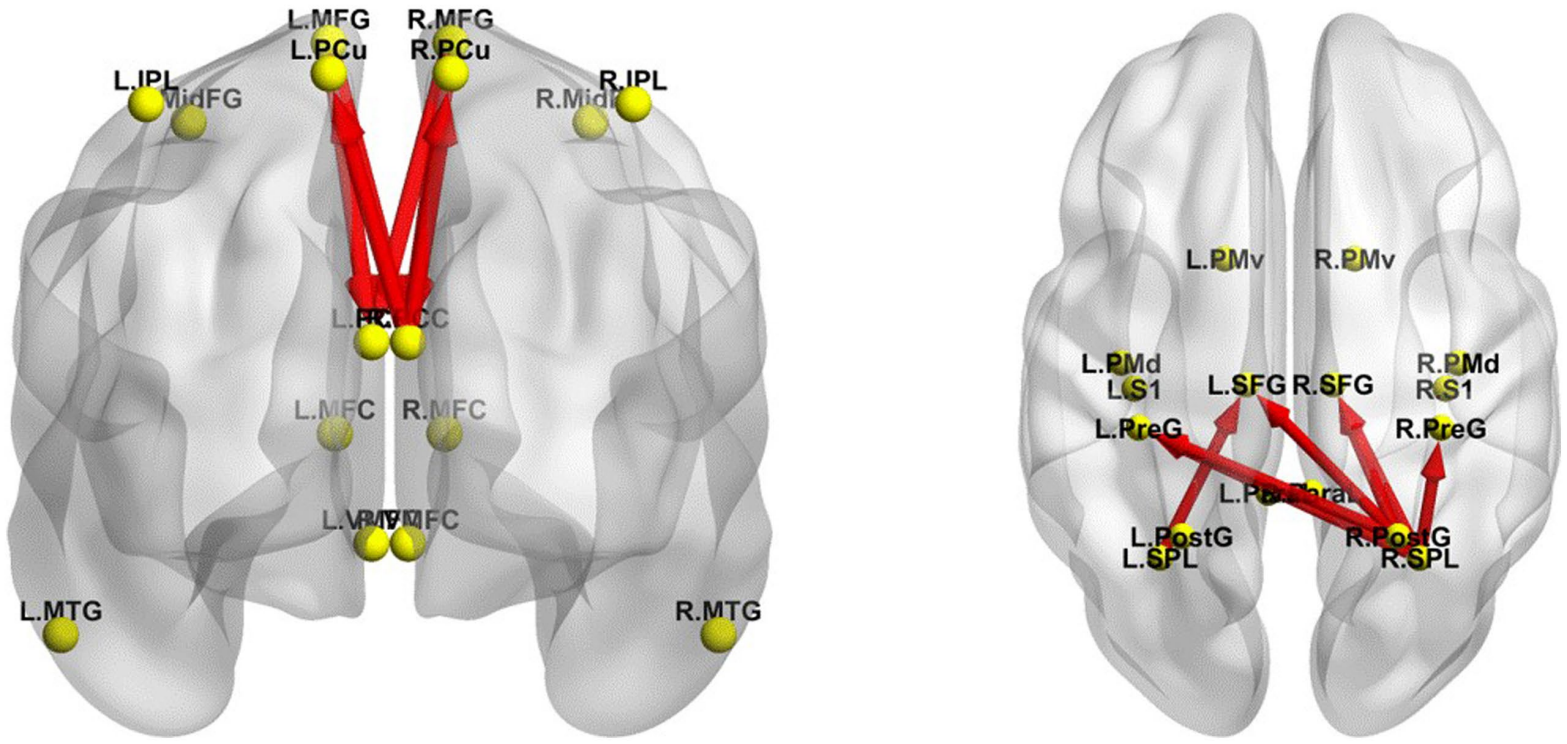
Changes in effective connectivity between EC and EO states in TSD. Left panel, DMN axial view (dorsal side); right panel, SMN coronal view (back side). Red indicates that the effective connectivity is higher in the EO state than in the EC state. The arrow indicates the direction of the connection. DMN, default mode network; EC, eyes closed; EO, eyes open; SMN, sensorimotor network; TSD, total sleep deprivation.

The iCoh results suggested that the effective connectivity in the DMN was significantly enhanced in EO compared with EC in TSD (*t-corrected* = 4.034, *p-corrected* = 0.0092) (Figure 7), including the effective connectivity from the left MFG to the left (*t-corrected* = 4.675) and right PCC (*t-corrected* = 4.277) in δ, θ, and α bands; from the right MFG to the left PCC (*t-corrected* = 4.501) in α and β bands and to the right PCC (*t-corrected* = 4.214) in α and β bands; from the right PCC to the left PCu (*t-corrected* = 4.188) in δ band, to the right PCu (*t-corrected* = 4.183) in δ and θ bands, and to the left PCC (*t-corrected* = 4.568) in δ, θ and α bands.

The iCoh results suggested that the effective connectivity in the SMN was significantly enhanced in EO compared with EC in TSD (*t-corrected* = 3.878, *p-corrected* < 0.001) (Figure 7), including the effective connectivity from the right SPL to right SFG in θ, α, β, and γ bands (*t-corrected* = 6.842), to the left SFG in α, β and γ bands (*t-corrected* = 6.813), to the right PreG in δ, θ, α, β, and γ bands (*t-corrected* = 5.783), to the left PreG in γ band (*t-corrected* = 4.159); from the right PostG to the right (*t-corrected* = 5.095) and left (*t-corrected* = 4.711) SFG in β, and γ bands, to the left PreG in γ band (*t-corrected* = 3.902); from the left SPL to the left SFG in β band (*t-corrected* = 3.909). Among all the frequency bands, the γ band was the most significant for above effective connectivity in SMN.

### Effective connectivity changes in EC or EO states between RW and TSD

**Figure 8:**
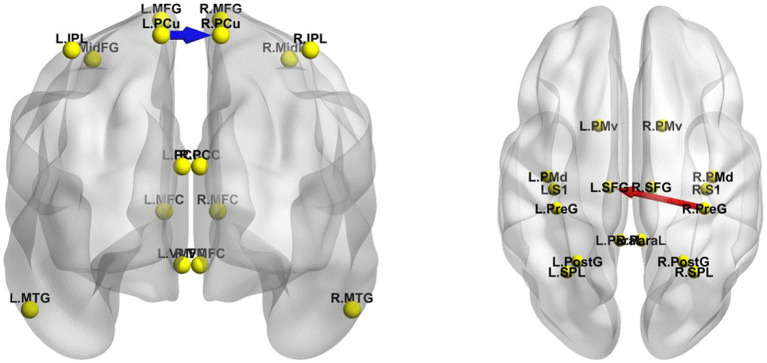
Changes in the effective connectivity of the DMN and SMN between RW and TSD. Left panel: DMN in the EC state, axial view (dorsal side); right panel: SMN in the EO state, coronal view (back side). Red indicates that the effective connectivity is higher in TSD than in RW; Blue indicates that the effective connectivity is lower in TSD than in RW. DMN, default mode network; EC, eyes closed; EO, eyes open; RW, rested wakefulness; SMN, sensorimotor network; TSD, total sleep deprivation.

The iCoh results suggested that in the EC state, the effective connectivity from the left PCu to the right PCu was significantly reduced in the β band after TSD (*t-corrected* = 4.041, *p-corrected* = 0.048) (Figure 8). Conversely, the effective connectivity from the right precentral gyrus to the left superior frontal gyrus was significantly enhanced in the β band after TSD in the EO state (*t-corrected* = 3.942, *p-corrected* = 0.036) (Figure 8).

### Effective connectivity changes in the different networks between RW and TSD

**Figure 9:**
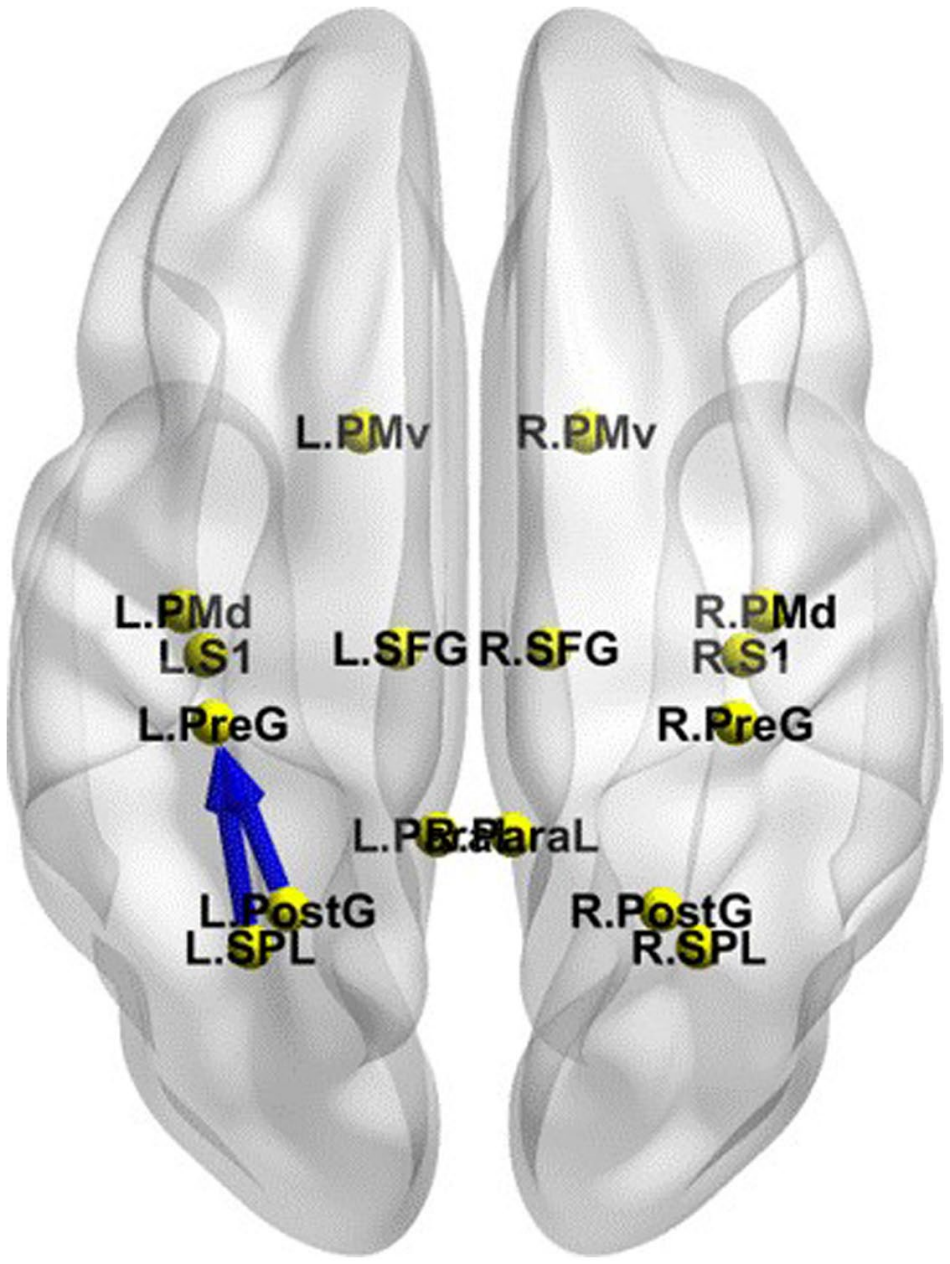
Changes in effective connectivity in the different networks (EO-EC) of SMN between RW and TSD. Blue indicates that the effective connectivity is lower in TSD than in RW. Coronal view (back side). EC, eyes closed; EO, eyes open; RW, rested wakefulness; SMN, sensorimotor network; TSD, total sleep deprivation.

The iCoh results suggested that the effective connectivity in the different networks (EO-EC) of the SMN was significantly decreased after TSD compared to RW (*t-corrected* = 4.046, *p-corrected* = 0.0016) (Figure 9). The left SPL sent to the left PreG oscillating at the β and γ bands (*t-corrected* = 3.5276, *p-corrected* < 0.049) and the left PostG sent to the left PreG oscillating at the γ band (*t-corrected* = 4.065, *p-corrected* < 0.049).

### Correlation analysis between the changes in effective connectivity and PVT RT

**Figure 10:**
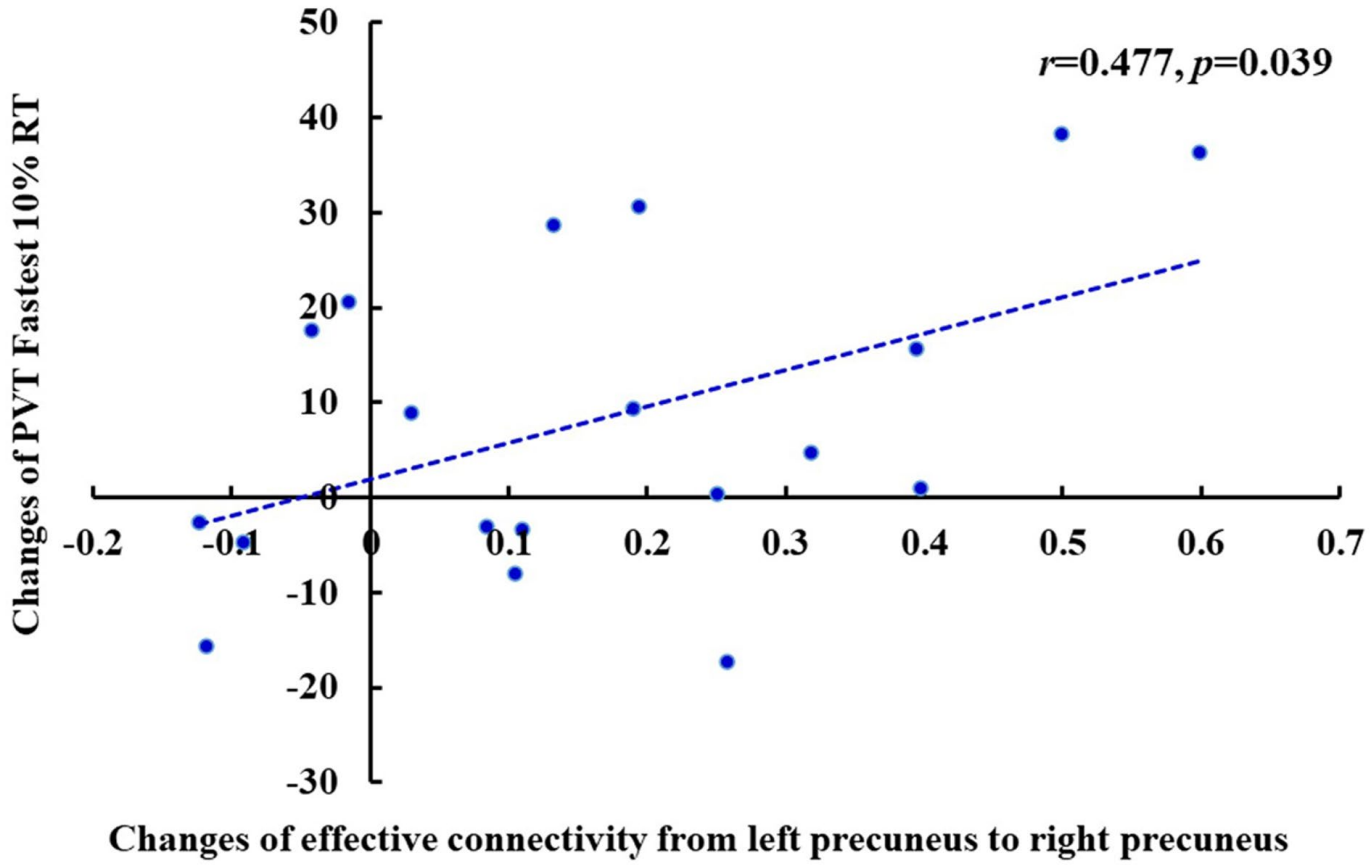
Changes of the effective connectivity from left precuneus to right precuneus after TSD in the EC state was positively correlated with changes of PVT Fastest 10% RT (ms) (*r* = 0.477, *p* = 0.039). EC, eyes closed; PVT, psychomotor vigilance task; TSD, total sleep deprivation.

**Figure 11:**
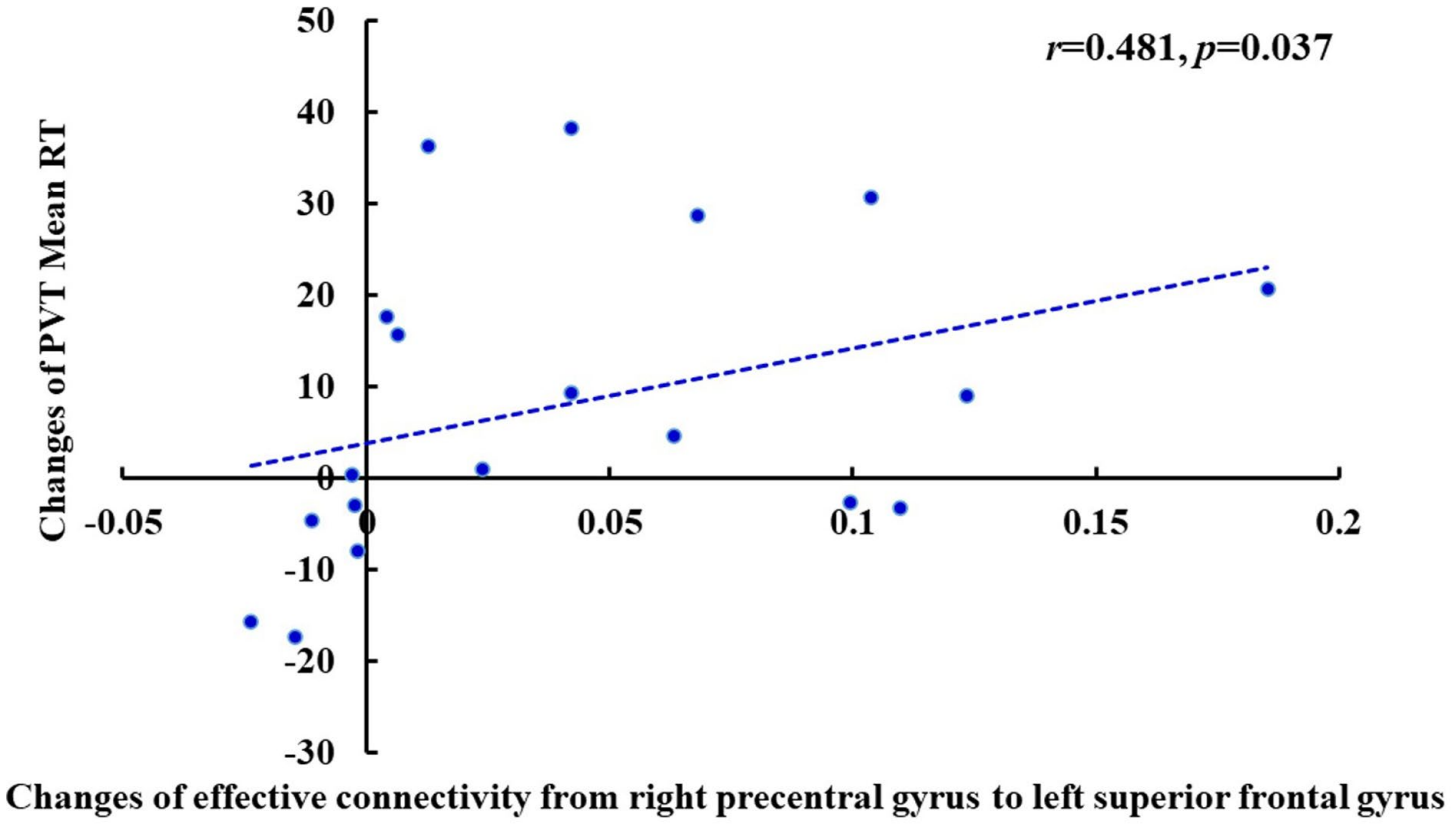
Changes of the effective connectivity from right precentral gyrus to left superior frontal gyrus after TSD in the EO state was positively correlated with changes of PVT Mean RT (ms) (*r* = 0.481, *p* = 0.037). EO, eyes open; PVT, psychomotor vigilance task; RT, reaction time; TSD, total sleep deprivation.

Pearson’s correlation analysis showed that after TSD, there was a significant positive correlation between the increase in the fastest 10% RT during the PVT and the decrease in the effective connectivity from the left PCu to the right PCu (*r* = 0.477, *p* = 0.039) of the DMN in the EC state (Figure 10). Furthermore, there was a significant positive correlation between the increase in the mean PVT RT and the enhancement in the effective connectivity from the right precentral gyrus to the left superior frontal gyrus (*r* = 0.481, *p* = 0.037) of the SMN in the EO state (Figure 11). However, after FDR correction, neither correlation was significant.

## Discussion

The current study compared brain activity within the DMN and SMN, in four conditions [two eye states (EC; EO) and two SD times (RW; TSD)], using resting-state EEG from both the spontaneous and synchronous perspectives. Our results indicate that TSD impairs alertness as well as sensory information input in the SMN, especially in the EO state, compared to those in the EC state.

### Differences in the DMN and SMN between the EC and EO states in both RW and TSD

Our study’s fICA and iCoh results showed that in RW, there was a tendency for brain activity changes in the EO state compared to those in the EC state; notably, there was an increase in high-frequency bands, and a decrease in low-frequency bands, for the DMN and SMN. Moreover, the iCoh results showed that in TSD, the effective connectivity in the EO state was stronger than that in the EC state.

In SMN, the neural oscillations in the postcentral gyrus and superior parietal lobule in the EO state were lower in both δ and θ bands and higher in both α and β bands than those in the EC state. Furthermore, the effective connectivity was enhanced in the α, β, and γ bands; this enhancement was most significant in the β band. Prior fMRI studies have demonstrated that activity in the SMN in the EO state is lower than that in the EC state. Liang et al. (2014) observed that compared to the EO state, the EC state induced higher fractional ALFF values in the sensation-related cortex. Furthermore, Liu et al. (2013) demonstrated that a striking similarity among the results of three different methods (ALFF, regional homogeneity, and seed-based correlation analysis) was evident for different eye states. This similarity included the increased visual, decreased primary sensorimotor, and auditory cortices in the EO state than in the EC state. Our study assessed the difference in spontaneous and synchronous activity in the SMN between EO and EC states from a space-frequency perspective, and we found that the decreased activity in the SMN in the EO state was mainly in the low-frequency bands (δ and θ), while the activity in high-frequency bands (α and β) increased. It was reported that the EO state induced lower δ and θ powers of the EEG spectra compared with the EC state (Allen et al., 2018). High-frequency activity is demonstrated to be associated with higher cognitive process (Deligianni et al., 2014; Fries, 2015; Sockeel et al., 2016), and increased alertness is accompanied by higher EEG activation in the β-band (Kamiński et al., 2012). Therefore, the higher activity of the SMN at high frequencies may be related to greater alertness and sensory information input during the EO state compared to those during the EC state.

Neural oscillations of the PCu in the DMN in the EO state were lower in both the δ and θ bands than those in the EC state. Furthermore, effective connectivity in the DMN was higher in the EO state in α, β, and γ bands compared with the EC state. This enhancement was most significant in the β band. Our study’s findings suggest significant differences in both the spontaneous and synchronous activity in the DMN between EC and EO states. This is consistent with previous studies (Yan et al., 2009). The higher activity of the DMN was mainly in the high-frequency (α and β) bands. In the DMN, the PCC is related to the general monitoring of sensory information (Raichle and Snyder, 2007). Furthermore, the PCu, MFG, and lateral parietal cortex have been demonstrated to be involved in episodic memory retrieval (Cavanna and Trimble, 2006). Moreover, the PCC and MFG have been reported to be associated with mind wandering and daydreaming (Mason et al., 2007). Therefore, the higher activity of the DMN in the EO state at high frequency may be due to increased gathering and monitoring of environmental information that caused more mind wandering or daydreaming compared with the EC state (Yan et al., 2009).

Previous studies have found inconsistent patterns of activity change between the EO and EC states in the DMN regions and the visual cortex (Yang et al., 2007; Liang et al., 2014). Our study’s findings indicate that these contradictory change patterns are associated with changes in frequency, with higher activity in EO states at high frequencies and lower activity in EO states at low frequencies.

Furthermore, in TSD, the effective connectivity in the EO state was found to be stronger than that in the EC state both at high and low frequencies. TSD is known to cause cognitive decline and a shortage of cognitive resources (Chee and Chuah, 2007; Simon et al., 2015; Lei et al., 2016). After TSD, microsleep was shown to intrude into wakefulness both in the EC and EO states owing to tremendous sleep pressure even though it was resisted by participants (Goel et al., 2009). However, the duration and frequency of microsleep are increased in the EC state compared to those in the EO state. Additionally, opening eyes could be an intentional behavior to mask the tendency to sleep to some extent, which reflects an antagonistic process exerted by individuals against sleepiness. Therefore, the higher effective connectivity in the EO state compared with that in the EC state in TSD may reflect participants’ efforts to combat sleep stress.

### Effect of TSD on the DMN and SMN in both EO and EC

In TSD, the activity of DMN was decreased, while SMN was increased, compared to those of RW. Moreover, the changed effective connectivity in the DMN and SMN after TSD was positively correlated with the increased PVT reaction time.

Several investigations have shown that SD results in lower activity in the DMN than RW and creates higher activity in the SMN. Several fMRI studies observed that SD induced significantly declined FC within the DMN in both resting states and visual attention task (Sämann et al., 2010; De Havas et al., 2012; Lei et al., 2015). Another fMRI study observed the functional connectivity densities (FCDs) at rest were lower in the DMN after TSD, and higher in the SMN. Besides, the declined FCD in the PCC was correlated with the increased RT of PVT after TSD (Yang et al., 2018). Cai et al. (2021) observed significantly lower ALFF of the DMN and frontal–parietal network after SD, and higher ALFF in the visual and motor cortex. Our study’s results regarding the DMN and SMN agree with those observed in previous studies.

The PCu is considered the core node of the DMN. It has been proposed that DMN mediated intrinsic activity after SD largely depends on the PCu and PCC with greater task-related deactivation (Fransson and Marrelec, 2008). Gujar et al. (2010) observed an imbalanced pattern of task-induced deactivation, with significantly less deactivation in the SD group within the dorsal anterior cingulate cortex region of the DMN yet significantly greater deactivation in the PCu. They also demonstrated that the imbalance was more indicative of dysfunction than compensatory effects, and accurately distinguished between sleep-deprived and normal people. Hence, we hypothesized that the decreased neural oscillation in the low-frequency band and decreased effective connectivity in the high-frequency band in the PCu suggested deficits in higher cognitive functions after TSD. Furthermore, that declined activity in the PCu might be a biomarker sensitive to indexing sleep loss.

Decreased visual-parietal FC after SD was observed by Fu et al. (2022) indicating the impaired function of processing environmental stimuli. SD can cause a continuous decrease in vigilant attention (Borragán et al., 2019; Qi et al., 2021). However, the brain compensates for this dysfunction, and this compensatory adaptation can partly offset impaired cognitive function (Drummond et al., 2000). Thus, the increased effective connectivity from the right precentral gyrus to the left superior frontal gyrus may compensate for drowsiness. The positive correlation with decreased PVT performance may indicate that the impaired alertness function was related to the blocked information gathering and disruption of the SMN.

The energy allocation model can also demonstrate our point of view that the limited cognitive resources after SD will be optimally allocated for survival (Schmidt, 2014). The DMN is mostly associated with some higher cognitive functions (Mason et al., 2007). Therefore, it is suppressed after TSD. However, activities of the SMN are vital to survival, with an important role in external information gathering, so the enhancement of SMN activity is considered to be the brain’s attempt to maintain alertness and attention to surroundings (Yang et al., 2018; Cai et al., 2021).

### Differences in the different networks (EO-EC) between TSD and RW

The current study indicated that the effective connectivity in the different networks (EO-EC) of the SMN in the β band was significantly decreased after TSD compared to RW. This indicates that TSD impaired alertness and sensory information input, especially in the EO state. Furthermore, in the different networks of the SMN, the effective connectivity from the left superior parietal lobule to the left precentral gyrus was decreased in the β and γ bands. Furthermore, the effective connectivity from the left postcentral gyrus to the left precentral gyrus was decreased in the γ band after TSD compared to RW. Nevertheless, no significant difference was observed in the neural oscillation of (EO-EC) between the RW and TSD.

The postcentral and precentral gyri are the main areas of the human brain’s motor and somatosensory cortices, respectively. The superior parietal lobule, which makes up a large portion of the parietal lobe, has many functions, such as audio-visual multisensory integration (Hawkins et al., 2013), transformation of sensory inputs into an appropriate motor response (Hecht et al., 2013), and visuospatial attention (Cai et al., 2013).

Additionally, many studies have indicated that β-activity is associated with attention arousal and sensorimotor function (Lalo et al., 2007; Kamiński et al., 2012; Gola et al., 2013). These decreased connections related to sensation and motor function in high-frequency bands jointly indicated that TSD disrupted resting SMN functions more in the EO state than in the EC state. This might cause a greater decline in environmental information input in the EO state. Decreased β activity can also be considered to be related to decreased alertness (Kamiński et al., 2012), and γ oscillations are associated with various cognitive activities, such as high alertness and attentional states (Theriault and Perreault, 2019). Usually, the EO state enhances participants’ level of alertness to their surroundings compared to the EC state. Thus, compared to RW, TSD might suppress information gathering, input process, and the level of alertness in the EO state as opposed to the EC state.

In summary, the SMN’s functions of alertness and collecting the surrounding environment’s information in the EO state were impaired after TSD with excessive drowsiness and limited psychological resources. The results demonstrated that alertness and sensory information input in the EO state were more impaired after TSD than those in the EC state. This implies that the EO state can better reflect the influence of TSD on cognitive function. Thus, it may be better to use an EO state rather than an EC state in future resting-state research.

### Limitations

The present study had some limitations. First, the study detected the effects of different eye states on the DMN and SMN. However, the interactions between different networks remain unclear. Second, given the partial overlap between different functional networks, other networks may have affected the selected network nodes. Third, because our experiment required participants to spend three nights in the lab, and we only had male nursing staff for continuous monitoring of participants, so, we did not recruit women, which might have induced bias in the results. It would be interesting to study gender differences in the effects of TSD on resting brain activity in the future. Fourth, the sample size was small. Fifth, the effective connectivity we measured is a form of static functional connectivity, but the internal mental activity of participants varies with time. Hence, future research should observe dynamic functional connectivity to identify the difference between EC and EO states. Sixth, the correlations between changes in effective connectivity and changes in PVT reaction time were not significant after multiple comparisons correction, which may be due to the small sample size. Additionally, the RW data were acquired at 8:00, while the TSD data were acquired at 20:00 on the next day. This might have mixed the effects of the circadian rhythm. Plus, the small sample size may also be a contributing factor. Thus, future studies should further investigate the differences in the interactions of networks between different eye states. In addition, the different effects of TSD on functional networks in different eye states should be further studied.

## Conclusion

This is the first study to explore the effects of TSD on resting-state brain activity in different eye states using eLORETA. The study showed that alertness and information input were higher in the EO state of RW than in the EC state. Besides, the limited energy was optimally allocated for survival-related functions within the SMN after TSD, and the higher cognitive functions within the DMN were suppressed. In addition, alertness and information input in the SMN were more impaired in the EO state of TSD than in the EC state. This implies that an EO state can better reflect the influence of TSD on cognitive function. Thus, employing an EO state rather than an EC state in future resting-state research may be better.

## Data availability statement

The raw data supporting the conclusions of this article will be made available by the authors, without undue reservation.

## Ethics statement

The studies involving humans were approved by the Biological and Medical Ethics Committee of Beihang University. The studies were conducted in accordance with the local legislation and institutional requirements. The participants provided their written informed consent to participate in this study.

## Author contributions

MM: conceptualization, methodology, formal analysis, data curation, writing—original draft preparation, and visualization. MM and YL: investigation. MM, YL, YS, and XW: writing—review and editing. YS and XW: supervision. All authors have read and agreed to the published version of the manuscript.

## References

[ref1] Allen E. A. Damaraju E. Eichele T. Wu L. Calhoun V. D. (2018). EEG signatures of dynamic functional network connectivity states. Brain Topogr. 31, 101–116. doi: 10.1007/s10548-017-0546-2, PMID: 28229308 PMC5568463

[ref2] Aoki Y. Ishii R. Pascual-Marqui R. D. Canuet L. Ikeda S. Hata M.. (2015). Detection of EEG-resting state independent networks by eLORETA-ICA method. Front. Hum. Neurosci. 9:31. doi: 10.3389/fnhum.2015.00031, PMID: 25713521 PMC4322703

[ref3] Babiloni C. Barry R. J. Basar E. Blinowska K. J. Cichocki A. Drinkenburg W.. (2020). International federation of clinical neurophysiology (IFCN) – EEG research workgroup: recommendations on frequency and topographic analysis of resting state EEG rhythms. Clin. Neurophysiol. 131, 285–307. doi: 10.1016/j.clinph.2019.06.234, PMID: 31501011

[ref4] Bembich S. Clarici A. Vecchiet C. Baldassi G. Cont G. Demarini S. (2014). Differences in time course activation of dorsolateral prefrontal cortex associated with low or high risk choices in a gambling task. Front. Hum. Neurosci. 8:464. doi: 10.3389/fnhum.2014.00464, PMID: 25009486 PMC4067729

[ref5] Berger H. (1930/1969). On the electroencephalogram of man: second report. Electroencephalogr. Clin. Neurophysiol. 28, 75–93. 4188919

[ref6] Berger H. (1931/1969). On the electroencephalogram of man: third report. Electroenceph. Clin. Neurophysiol. 28, 95–132. 4188920

[ref7] Borragán G. Guerrero-Mosquera C. Guillaume C. Slama H. Peigneux P. (2019). Decreased prefrontal connectivity parallels cognitive fatigue-related performance decline after sleep deprivation: an optical imaging study. Biol. Psychol. 144, 115–124. doi: 10.1016/j.biopsycho.2019.03.004, PMID: 30930071

[ref8] Buysse D. J. Reynolds C. F. III Monk T. H. Berman S. R. Kupfer D. J. (1989). The Pittsburgh sleep quality index: a new instrument for psychiatric practice and research. Psychiatry Res. 28, 193–213. doi: 10.1016/0165-1781(89)90047-4, PMID: 2748771

[ref9] Cabeza R. Dolcos F. Graham R. Nyberg L. (2002). Similarities and differences in the neural correlates of episodic memory retrieval and working memory. Neuroimage 16, 317–330. doi: 10.1006/nimg.2002.1063, PMID: 12030819

[ref10] Cai Y. Mai Z. Li M. Zhou X. Ma N. (2021). Altered frontal connectivity after sleep deprivation predicts sustained attentional impairment: a resting-state functional magnetic resonance imaging study. J. Sleep Res. 30:e13329. doi: 10.1111/jsr.13329, PMID: 33686744

[ref11] Cai Q. Van L. Brysbaert M. (2013). Complementary hemispheric specialization for language production and visuospatial attention. Proc. Natl. Acad. Sci. U. S. A. 110, 322–330. doi: 10.1073/pnas.1212956110 PMC3557046 23297206

[ref12] Cavanna A. E. Trimble M. R. (2006). The precuneus: a review of its functional anatomy and behavioural correlates. Brain 129, 564–583. doi: 10.1093/brain/awl004, PMID: 16399806

[ref13] Charroud C. Steffener J. Le Bars E. Deverdun J. Bonafe A. Abdennour M.. (2015). Working memory activation of neural networks in the elderly as a function of information processing phase and task complexity. Neurobiol. Learn. Mem. 125, 211–223. doi: 10.1016/j.nlm.2015.10.002, PMID: 26456114

[ref14] Chee M. W. L. Chuah Y. M. L. (2007). Functional neuroimaging and behavioral correlates of capacity decline in visual short-term memory after sleep deprivation. Proc. Natl. Acad. Sci. U. S. A. 104, 9487–9492. doi: 10.1073/pnas.0610712104, PMID: 17517619 PMC1874228

[ref15] Chen A. C. Feng W. Zhao H. Yin Y. Wang P. (2008). EEG default mode network in the human brain: spectral regional field powers. Neuroimage 41, 561–574. doi: 10.1016/j.neuroimage.2007.12.064, PMID: 18403217

[ref16] Chen J. L. Ros T. Gruzelier J. H. (2013). Dynamic changes of ICA-derived EEG functional connectivity in the resting state. Hum. Brain Mapp. 34, 852–868. doi: 10.1002/hbm.21475 22344782 PMC6870341

[ref17] Chiang H. S. Pao S. C. (2016). An EEG-based fuzzy probability model for early diagnosis of Alzheimer’s disease. J. Med. Syst. 40:125. doi: 10.1007/s10916-016-0476-7, PMID: 27059738

[ref18] Christoff K. Gordon A. M. Smallwood J. Smith R. Schooler J. W. (2009). Experience sampling during fMRI reveals default network and executive system contributions to mind wandering. Proc. Natl. Acad. Sci. U. S. A. 106, 8719–8724. doi: 10.1073/pnas.0900234106, PMID: 19433790 PMC2689035

[ref19] De Havas J. A. Parimal S. Soon C. S. Chee M. W. L. (2012). Sleep deprivation reduces default mode network connectivity and anti-correlation during rest and task performance. Neuroimage 59, 1745–1751. doi: 10.1016/j.neuroimage.2011.08.026, PMID: 21872664

[ref20] Deligianni F. Centeno M. Carmichael D. W. Clayden J. D. (2014). Relating resting-state fMRI and EEG whole-brain connectomes across frequency bands. Front. Neurosci. 8:258. doi: 10.3389/fnins.2014.00258, PMID: 25221467 PMC4148011

[ref21] Dorrian J. Rogers N. L. Dinges D. F. Kushida C. A. (2005). “Psychomotor vigilance performance: neurocognitive assay sensitive to sleep loss” in Sleep deprivation: clinical issues, pharmacology and sleep loss effects. ed. Kushida C. A. (New York, NY: Marcel Dekker, Inc), 39–70.

[ref22] Drummond S. P. Brown G. G. Gillin J. C. Stricker J. L. Wong E. C. Buxton R. B. (2000). Altered brain response to verbal learning following sleep deprivation. Nature 403, 655–657. doi: 10.1038/35001068, PMID: 10688201

[ref23] Fox M. D. Snyder A. Z. Vincent J. L. Corbetta M. Van Essen D. C. Raichle M. E. (2005). The human brain is intrinsically organized into dynamic, anticorrelated functional networks. Proc. Natl. Acad. Sci. U. S. A. 102, 9673–9678. doi: 10.1073/pnas.0504136102, PMID: 15976020 PMC1157105

[ref24] Fransson P. (2005). Spontaneous low-frequency BOLD signal fluctuations: an fMRI investigation of the resting-state default mode of brain function hypothesis. Hum. Brain Mapp. 26, 15–29. doi: 10.1002/hbm.20113, PMID: 15852468 PMC6871700

[ref25] Fransson P. Marrelec G. (2008). The precuneus/posterior cingulate cortex plays a pivotal role in the default mode network: evidence from a partial correlation network analysis. Neuroimage 42, 1178–1184. doi: 10.1016/j.neuroimage.2008.05.059, PMID: 18598773

[ref26] Fries P. (2015). Rhythms for cognition: communication through coherence. Neuron 88, 220–235. doi: 10.1016/j.neuron.2015.09.034 26447583 PMC4605134

[ref27] Fu W. Dai C. Chen J. Wang L. Song T. Peng Z.. (2022). Altered insular functional connectivity correlates to impaired vigilant attention after sleep deprivation: a resting-state functional magnetic resonance imaging study. Front. Neurosci. 16:889009. doi: 10.3389/fnins.2022.889009, PMID: 35958999 PMC9361853

[ref28] Fuchs M. Kastner J. Wagner M. Hawes S. Ebersole J. S. (2002). A standardized boundary element method volume conductor model. Clin. Neurophysiol. 113, 702–712. doi: 10.1016/S1388-2457(02)00030-5, PMID: 11976050

[ref29] Goel N. Rao H. Durmer S. Dinges D. (2009). Neurocognitive consequences of sleep deprivation. Semin. Neurol. 29, 320–339. doi: 10.1055/s-0029-1237117 19742409 PMC3564638

[ref30] Gola M. Magnuski M. Szumska I. Wróbel A. (2013). EEG beta band activity is related to attention and attentional deficits in the visual performance of elderly subjects. Int. J. Psychophysiol. 89, 334–341. doi: 10.1016/j.ijpsycho.2013.05.007, PMID: 23688673

[ref31] Gujar N. Yoo S.-S. Hu P. Walker M. P. (2010). The unrested resting brain: sleep deprivation alters activity within the de-fault-mode network. J. Cogn. Neurosci. 22, 1637–1648. doi: 10.1162/jocn.2009.21331, PMID: 19702469 PMC2883887

[ref32] Gusnard D. A. Raichle M. E. (2001). Searching for a baseline: functional imaging and the resting human brain. Nat. Rev. Neurosci. 2, 685–694. doi: 10.1038/35094500, PMID: 11584306

[ref33] Hawkins K. M. Sayegh P. Yan X. Crawford J. D. Sergio L. E. (2013). Neural activity in superior parietal cortex during rule-based visual motor transformations. J. Cogn. Neurosci. 25, 436–454. doi: 10.1162/jocn_a_00318, PMID: 23092356

[ref34] Hecht E. E. Gutman D. A. Preuss T. M. Sanchez M. M. Parr L. A. Rilling J. K. (2013). Process versus product in social learning: comparative diffusion tensor imaging of neural systems for action execution–observation matching in macaques, chimpanzees, and humans. Cereb. Cortex 23, 1014–1024. doi: 10.1093/cercor/bhs097, PMID: 22539611 PMC3615349

[ref35] Hertig-Godeschalk A. Skorucak J. Malafeev A. Achermann P. Mathis J. Schreier D. R. (2020). Microsleep episodes in the borderland between wakefulness and sleep. SLEEPJ 43:zsz163. doi: 10.1093/sleep/zsz163, PMID: 31328230

[ref36] Japee S. Holiday K. Satyshur M. D. Mukai I. Ungerleider L. G. (2015). A role of right middle frontal gyrus in reorienting of attention: a case study. Front. Syst. Neurosci. 9:23. doi: 10.3389/fnsys.2015.00023, PMID: 25784862 PMC4347607

[ref37] Kamiński J. Brzezicka A. Gola M. Wróbel A. (2012). Beta band oscillations engagement in human alertness process. Int. J. Psychophysiol. 85, 125–128. doi: 10.1016/j.ijpsycho.2011.11.006, PMID: 22155528

[ref38] Kozasa E. H. Sato J. R. Russell T. A. Barreiros M. A. Lacerda S. S. Radvany J.. (2017). Differences in default mode network connectivity in meditators and non-meditators during an attention task. J. Cogn. Enhan. 1, 228–234. doi: 10.1007/s41465-017-0031-6

[ref39] Kucyi A. Salomons T. V. Davis K. D. (2013). Mind wandering away from pain dynamically engages antinociceptive and default mode brain networks. Proc. Natl. Acad. Sci. U. S.A. 110, 18692–18697. doi: 10.1073/pnas.1312902110, PMID: 24167282 PMC3832014

[ref40] Lalo E. Gilbertson T. Doyle L. Di Lazzaro V. Cioni B. Brown P. (2007). Phasic increases in cortical beta activity are as-sociated with alterations in sensory processing in the human. Exp. Brain Res. 177, 137–145. doi: 10.1007/s00221-006-0655-8, PMID: 16972074

[ref41] Lei Y. Shao Y. Wang L. Zhai T. Zou F. Ye E.. (2015). Large-scale brain network coupling predicts total sleep deprivation effects on cognitive capacity. PLoS One 10:e0133959. doi: 10.1371/journal.pone.0133959, PMID: 26218521 PMC4517902

[ref42] Lei Y. Wang L. Chen P. Li Y. Han W. Ge M.. (2016). Neural correlates of increased risk-taking propensity in sleep-deprived people along with a changing risk level. Brain Imaging Behav. 11, 1910–1921. doi: 10.1007/s11682-016-9658-7, PMID: 27975159

[ref43] Liang B. Zhang D. Wen X. Xu P. Peng X. Huang X.. (2014). Brain spontaneous fluctuations in sensorimotor regions were directly related to eyes open and eyes closed: evidences from a machine learning approach. Front. Hum. Neurosci. 8:645. doi: 10.3389/fnhum.2014.00645 25191258 PMC4138937

[ref44] Lim J. Dinges D. F. (2008). Sleep deprivation and vigilant attention. Ann. N. Y. Acad. Sci. 1129, 305–322. doi: 10.1196/annals.1417.002 18591490

[ref45] Liu D. Dong Z. Zuo X. Wang J. Zang Y. (2013). Eyes-open/eyes-closed dataset sharing for reproducibility evaluation of resting state fMRI data analysis methods. Neuroinformatics 11, 469–476. doi: 10.1007/s12021-013-9187-0, PMID: 23836389

[ref46] Liu X. Wu X. Zhong M. Huang H. Weng Y. Niu M.. (2020). Dynamic properties of human default mode network in eyes-closed and eyes-open. Brain Topogr. 33, 720–732. doi: 10.1007/s10548-020-00792-3, PMID: 32803623

[ref47] Marx E. Deutschländer A. Stephan T. Dieterich M. Wiesmann M. Brandt T. (2004). Eyes open and eyes closed as rest conditions: impact on brain activation patterns. Neuroimage 21, 1818–1824. doi: 10.1016/j.neuroimage.2003.12.026, PMID: 15050602

[ref48] Marx E. Stephan T. Nolte A. Deutschländer A. Seelos K. C. Dieterich M.. (2003). Eye closure in darkness animates sensory systems. Neuroimage 19, 924–934. doi: 10.1016/S1053-8119(03)00150-2, PMID: 12880821

[ref49] Mason M. F. Norton M. I. Van Horn J. D. Wegner D. M. Grafton S. T. Macrae C. N. (2007). Wandering minds: the default network and stimulus-independent thought. Science 315, 393–395. doi: 10.1126/science.1131295, PMID: 17234951 PMC1821121

[ref50] Mazziotta J. Toga A. Evans A. Fox P. Lancaster J. Zilles K.. (2001). A probabilistic atlas and reference system for the human brain: international consortium for brain mapping (ICBM). Philos Trans R Soc Lond B Biol Sci. 356, 1293–1322. doi: 10.1098/rstb.2001.0915, PMID: 11545704 PMC1088516

[ref51] Morrone E. Lupo N. Trentin R. Pizza F. Risi I. Arcovio S.. (2019). Microsleep as a marker of sleepiness in obstructive sleep apnea patients. J. Sleep Res. 2019:e12882. doi: 10.1111/jsr.12882 31180173

[ref52] Painold A. Faber P. L. Reininghaus E. Z. Mörkl S. Holl A. K. Achermann P.. (2020). Reduced brain electric activity and functional connectivity in bipolar euthymia: an SLORETA source localization study. Clin. EEG Neurosci. 51, 155–166. doi: 10.1177/1550059419893472, PMID: 31845595

[ref53] Park C. H. Choi Y. S. Kim H. J. Chung H. K. Jung A. R. Yoo J. H.. (2018). Interactive effects of seizure frequency and lateralization on intratemporal effective connectivity in temporal lobe epilepsy. Epilepsia 59, 215–225. doi: 10.1111/epi.13951, PMID: 29205291

[ref54] Pascual-Marqui R. D. (2002). Standardized low-resolution brain electromagnetic tomography (sLORETA): technical details. Methods Find. Exp. Clin. Pharmacol. 24, 5–12. 12575463

[ref55] Pascual-Marqui R. D. (2007). Discrete, 3D distributed linear imaging methods of electric neuronal activity. Part 1: exact, zero error localization. Available at: http://arxiv.org/pdf/0710.3341

[ref56] Pascual-Marqui R. D. Biscay R. J. Bosch-Bayard J. Lehmann D. Kochi K. Kinoshita T.. (2014). Assessing direct paths of intracortical causal information flow of oscillatory activity with the isolated effective coherence (iCoh). Front. Hum. Neurosci. 8:448. doi: 10.3389/fnhum.2014.00448, PMID: 24999323 PMC4064566

[ref57] Pascual-Marqui R. D. Biscay-Lirio R. J. (2011). Interaction patterns of brain activity across space, time and frequency. Part I: methods. ar Xiv: 1103.2852v2[stat.ME]. Available at: http://arxiv.org/abs/1103.2852 (Accessed on March 15, 2011).

[ref58] Patriat R. Molloy E. K. Meier T. B. Kirk G. R. Nair V. A. Meyerand M. E.. (2013). The effect of resting condition on resting-state fMRI reliability and consistency: a comparison between resting with eyes open, closed, and fixated. Neuroimage 78, 463–473. doi: 10.1016/j.neuroimage.2013.04.013, PMID: 23597935 PMC4003890

[ref59] Qi J. Li B. Z. Zhang Y. Pan B. Gao Y. H. Zhan H.. (2021). Altered insula-prefrontal functional connectivity correlates to decreased vigilant attention after total sleep deprivation. Sleep Med. 84, 187–194. doi: 10.1016/j.sleep.2021.05.037, PMID: 34166985

[ref60] Raichle M. E. (2006). The brain’s dark energy. Science 314, 1249–1250. doi: 10.1126/science.1134405 PMID: 17124311

[ref61] Raichle M. E. Snyder A. Z. (2007). A default mode of brain function: a brief history of an evolving idea. Neuroimage 37, 1083–1090 (discussion 1097–1089). doi: 10.1016/j.neuroimage.2007.02.041, PMID: 17719799

[ref62] Sämann P. G. Tully C. Spoormaker V. I. Wetter T. C. Holsboer F. Wehrle R.. (2010). Increased sleep pressure reduces resting state functional connectivity. Magma 23, 375–389. doi: 10.1007/s10334-010-0213-z 20473549

[ref63] Satpute A. B. Lindquist K. A. (2019). The default mode network’s role in discrete emotion. Trends Cogn. Sci. 23, 851–864. doi: 10.1016/j.tics.2019.07.003, PMID: 31427147 PMC7281778

[ref64] Schmidt M. H. (2014). The energy allocation function of sleep: a unifying theory of sleep, torpor, and continuous wakefulness. Neurosci. Biobehav. Rev. 47C, 122–153. doi: 10.1016/j.neubiorev.2014.08.001 25117535

[ref65] Shirer W. R. Ryali S. Rykhlevskaia E. Menon V. Greicius M. D. (2012). Decoding subject-driven cognitive states with whole-brain connectivity patterns. Cereb. Cortex 22, 158–165. doi: 10.1093/cercor/bhr099, PMID: 21616982 PMC3236795

[ref66] Simon E. B. Oren N. Sharon H. Kirschner A. Goldway N. Okon-Singer H.. (2015). Losing neutrality: the neural basis of impaired emotional control without sleep. J. Neurosci. 35, 13194–13205. doi: 10.1523/JNEUROSCI.1314-15.2015, PMID: 26400948 PMC6605430

[ref67] Simpson J. R. Jr. Drevets W. C. Snyder A. Z. Gusnard D. A. Raichle M. E. (2001). Emotion-induced changes in human medial prefrontal cortex: II. During anticipatory anxiety. Proc. Natl. Acad. Sci. U. S. A. 98, 688–693. doi: 10.1073/pnas.98.2.688, PMID: 11209066 PMC14649

[ref68] Snyder S. M. Hall J. R. Cornwell S. L. Falk J. D. (2011). Addition of EEG improves accuracy of a logistic model that uses neuropsychological and cardiovascular factors to identify dementia and MCI. Psychiatry Res. 186, 97–102. doi: 10.1016/j.psychres.2010.04.058, PMID: 20817309

[ref69] Sockeel S. Schwartz D. Pélégrini-Issac M. Benali H. (2016). Large-scale functional networks identified from resting-state EEG using spatial ICA. PLoS One 11:e0146845. doi: 10.1371/journal.pone.0146845, PMID: 26785116 PMC4718524

[ref70] Thatcher R. W. North D. Biver C. (2005). Parametric vs. non-parametric statistics of low resolution electromagnetic tomography (LORETA). Clin. EEG Neurosci. 36, 1–8. doi: 10.1177/155005940503600103, PMID: 15683191

[ref71] Theriault R. Perreault M. L. (2019). Hormonal regulation of circuit function: sex, systems and depression. Biol. Sex Differ. 10:12. doi: 10.1186/s13293-019-0226-x, PMID: 30819248 PMC6394099

[ref72] Van Dijk K. R. Hedden T. Venkataraman A. Evans K. C. Lazar S. W. Buckner R. L. (2010). Intrinsic functional connectivity as a tool for human connectomics: theory, properties, and optimization. Neurophysiology 103, 297–321. doi: 10.1152/jn.00783.2009, PMID: 19889849 PMC2807224

[ref73] Wang X. Li J. Wang M. Yuan Y. Zhu L. Shen Y.. (2018). Alterations of the amplitude of low-frequency fluctuations in anxiety in Parkinson’s disease. Neurosci. Lett. 668, 19–23. doi: 10.1016/j.neulet.2018.01.010, PMID: 29309855

[ref74] Xu P. Huang R. Wang J. Van Dam N. T. Xie T. Dong Z.. (2014). Different topological organization of human brain functional networks with eyes open versus eyes closed. Neuroimage 90, 246–255. doi: 10.1016/j.neuroimage.2013.12.060, PMID: 24434242

[ref75] Yan C. Liu D. He Y. Zou Q. Zhu C. Zuo X.. (2009). Spontaneous brain activity in the default mode network is sensitive to different resting-state conditions with limited cognitive load. PLoS One 4:e5743. doi: 10.1371/journal.pone.0005743, PMID: 19492040 PMC2683943

[ref76] Yang L. Lei Y. Wang L. Chen P. Cheng S. Chen S.. (2018). Abnormal functional connectivity density in sleep-deprived subjects. Brain Imaging Behav. 12, 1650–1657. doi: 10.1007/s11682-018-9829-9, PMID: 29488149

[ref77] Yang H. Long X. Y. Yang Y. H. Yan H. Zhu C. Z. Zhou X. P.. (2007). Amplitude of low frequency fluctuation within visual areas revealed by resting-state functional MRI. Neuroimage 36, 144–152. doi: 10.1016/j.neuroimage.2007.01.054, PMID: 17434757

[ref78] Yuan B. Wang J. Zang Y. Liu D. (2014). Amplitude differences in high-frequency fMRI signals between eyes open and eyes closed resting states. Front. Hum. Neurosci. 8:503. doi: 10.3389/fnhum.2014.00503 25071530 PMC4086401

[ref79] Zhou X. Lei X. (2018). Wandering minds with wandering brain networks. Neurosci. Bull. 34, 1017–1028. doi: 10.1007/s12264-018-0278-7, PMID: 30136075 PMC6246840

